# The effects of modest drinking on life expectancy and mortality risks: a population-based cohort study

**DOI:** 10.1038/s41598-022-11427-x

**Published:** 2022-05-06

**Authors:** Yen-Tze Liu, June Han Lee, Min Kuang Tsai, James Cheng-Chung Wei, Chi-Pang Wen

**Affiliations:** 1 Department of Family Medicine, Changhua Christian Hospital, Changhua, Taiwan, ROC; 2 Institute of Medicine, Chung Shan Medical University, Taichung, Taiwan, ROC; 3 Department of Holistic Wellness, Mingdao University, Changhua, Taiwan, ROC; 4 Department of Post-Baccalaureate Medicine, National Chung Hsing University, Taichung, Taiwan, ROC; 5 National Health Research Institutes, Zhunan, Miaoli County Taiwan, ROC; 6 Department of Medicine, Chung Shan Medical University Hospital, Taichung, Taiwan, ROC; 7 Graduate Institute of Integrated Medicine, China Medical University, Taichung, Taiwan, ROC; 8 China Medical University, Taichung, Taiwan, ROC

**Keywords:** Diseases, Health care, Medical research, Risk factors

## Abstract

Modest drinking has been repeatedly discussed in scientific papers as protective against certain diseases, such as cardiovascular diseases, but in most cases, alcohol worsens health conditions, especially when consumed at high risk levels. The complexity of the risk relationship between alcohol and health conditions has confused clinicians as to whether it should be recommended. The study aims to balance the risks and benefits of modest drinking. This retrospective cohort study of 430,016 adults recruited from a standard health-screening program since 1994, with 11,031 deaths identified as of 2008. Drinking distinguished “modest drinker” (no more than one drink a day) from “regular drinker”. Mortality risks including all-cause mortality and diseases-specific mortality with hazard ratio (HR) were calculated by adjusting for 15 confounders. Life table was used for life expectancy. Risk predictors were subjected to Cox proportional hazards regression analysis to identify significant predictors in multivariate models and life expectancy analysis. Nearly one out of 4 males (23%) was a modest drinker, who gained 0.94 year (95% CI 0.65–1.23 year) in life over non-drinker and had 8% reduction in adjusted all-cause mortality (HR 0.92, 95% CI 0.86–0.97). In contrast, regular drinkers had 43% increase in overall mortality (HR 1.43, CI 1.35–1.52) and shortened life by 6.9 years (95% CI 6.6–7.1 years). As most drinkers also smoked, 59% in modest and 75% in regular, the combined effect shortened life by 2.0 years (95% CI 1.6–2.4 years) in modest drinker and 10.3 years (95% CI 9.8–10.7 years) in regular drinker. Cancer were increased in modest drinkers for oral (HR 2.35, CI 1.38–4.01) and esophageal (HR 3.83, CI 1.90–7.73) cancer. The gain of one year by modest drinkers was erased by a two to fourfold increase in oral and esophageal cancer and that drinking beyond modest amount led to a large loss of life expectancy. Given that drinkers are prone to cross the line of drinking, clinicians should balance the risks and benefits of drinking, as well as the understanding of whether the patient is at risk for addiction.

## Introduction

Drinking alcohol appears to have a complex and controversial relationship with health outcome. The “J-shaped” relationship between drinking and mortality has been well cited in the scientific literature^1–3^, but the concept and practice not yet fully understood by the public. A recurring theme is that although high doses of alcohol are harmful to the heart and causes cancer of multiple sites along the gastrointestinal tract (oral, esophagus, liver, and colorectal cancer), breast and larynx, mild to modest alcohol consumption has been associated with reductions in coronary artery diseases, diabetes, and even all-cause mortality^4^. In several systematic reviews and meta-analyses, modest alcohol consumption was found to be beneficial for various disease mortality and incidence risks^4–12^, especially in reducing cardiovascular disease (CVD) mortality and coronary heart disease incidence and mortality^13–16^. Furthermore, several epidemiological theories have been proposed to criticize the benefits of modest drinking, including abstainer bias, insufficient confounding factors adjustment, and “sick quitter hypothesis” means that individuals stop drinking due to serious illness^17^. The health outcome related to modest alcohol intake continues to be debated until now.

An important meta-analysis of 34 prospective cohort with one million subjects found 1–2 drinks for women and 2–4 drinks for men inversely associated with total mortality, with an estimated 16–19% benefit^18^. In other words, the cumulative evidence is that modest drinkers had lower all-cause mortality and survived better than never drinkers, even after confounders were considered. A more recent review of 83 prospective cohorts also found consuming alcohol at 100 g/week (around one drink a day) with the lowest cardiovascular disease risks^19^. Limited to the studying of 600,000 drinkers, authors in this study justified their report overlooking or not mentioning the risk of non-drinker. However, it was pointed out that data found in that study indicated a hidden 20% higher risk among non-drinker than the modest drinkers. The results were consistent with earlier studies but the concept of modest drinking is novel and against main stream thinking to accept “a little drinking is better than no drinking”. Furthermore, this review indicated that the all-cause mortality and hear failure risk were positively association with alcohol consumption^19^. However, other studies have suggested a J-type relationship between these two and alcohol consumption^4,6^. This demonstrates the complexity and inconclusive findings of past studies on the topic of modest drinking.

Mortality reduction between 16 and 20% may look simply to cheer about, but it is actually difficult for the public to appreciate its magnitude. As a relative risk, 20% could be high or low depending on which reference group was used. As described in the meta-analysis^18^, the results may vary depending on the subjects included in the reference group, and the protective effect was reduced when ex-drinkers and light drinkers were excluded from the reference group. Comparison of relative risk is difficult and could be misleading. Another problem is whether a hidden trade-off was involved in that accepting benefits are associated with a possibility of crossing the line of drinking into excess, where a much larger increase in mortality, like 50% or 100%, maybe involved. In this study, in addition to the use of relative risks, we used absolute risk (life expectancy), and tried to present a balanced view of both sides of the issue. Life expectancy is an absolute risk that is well understood by the general public. The public may be vaguely aware that alcohol consumption may be harmful to health, but to date there is a lack of reports of its effects on life expectancy in modest drinkers.

Another controversy surrounding what constituted the amount in modest drinking. In the U.S. and Australia, the drinking recommendation used to be up to 2 drinks for men and 1 drink for women, with an acceptable risk of one death per 100 people in their lifetime. However, in the last 2–3 years, both the US and UK called for lowering the amount from 2 to 4 drinks to one drink a day^20–22^. However, changes in the official recommendation are not always well known to consumers, and physicians may be confused about the definition of “modest” when conducting nutritional health counseling.

With the availability of a large cohort of nearly half a million subjects followed between 1996 and 2008, we compared modest drinkers (no more than one drink a day) or regular drinkers with non-drinker. Because a large number of drinkers also smoke, we analyzed the risks individually and in combinations.

## Results

During study period, 430,016 participants were analyzed. Table 1 shows the distribution of the demographic characteristics of the MJ Health Screening Center (MJ) cohort by drinking status. There were 339,267 (78.8%) participants without drinking, 60,309 (14.0%) modest drinkers, and 30,440 (7.1%) regular drinkers. It should be noted that the regular drinkers in Table 1 includes the ex-drinker group because the portion of ex-drinker was very small (3%) but the hazard ratios were large and were comparable with regular drinkers. The result of ex-drinker was shown in the Table S1 and S2. Modest drinkers were more educated, less obese, more active, less smoked, and had lower rates of hypertension, diabetes, and high triglycerides, proteinuria, high uric acid and high level of C-reactive protein when compared with regular drinkers. In addition, male and female demographics and clinical characteristics by drinking status presented separately in Table S3 and S4 as the difference of health risk in relation to alcohol between male and females does exist.

**Table 1:** Demographics and clinical characteristics by drinking status.

	Total		Non-drinker		Modest drinker						Regular drinker^§^						*P* value
							Never smoker		Smoker				Never smoker		Smoker		
	n	(%)	n	(%)	n	(%)	n	(%)	n	(%)	n	(%)	n	(%)	n	(%)	
Total	430,016	(100.0)	339,267	(78.9)	60,309	(14.0)	24,507	(5.7)	35,802	(8.3)	30,440	(7.1)	7658	(1.8)	22,782	(5.3)	
**Age**																	< 0.001
20–39	243,723	(100.0)	199,709	(81.9)	30,478	(12.5)	11,382	(4.7)	19,096	(7.8)	13,536	(5.6)	2780	(1.1)	10,756	(4.4)	
40–64	158,227	(100.0)	118,394	(74.8)	25,728	(16.3)	11,659	(7.4)	14,069	(8.9)	14,105	(8.9)	4098	(2.6)	10,007	(6.3)	
≧ 65	28,066	(100.0)	21,164	(75.4)	4103	(14.6)	1466	(5.2)	2637	(9.4)	2799	(10.0)	780	(2.8)	2019	(7.2)	
**Gender**																	< 0.001
Male	210,594	(100.0)	137,664	(65.4)	47,540	(22.6)	14,999	(7.1)	32,541	(15.5)	25,390	(12.1)	5303	(2.5)	20,087	(9.5)	
Female	219,422	(100.0)	201,603	(91.9)	12,769	(5.8)	9508	(4.3)	3261	(1.5)	5050	(2.3)	2355	(1.1)	2695	(1.2)	
**Education**																	< 0.001
≦ Middle school	102,975	(100.0)	77,218	(75.0)	15,328	(14.9)	6668	(6.5)	8660	(8.4)	10,429	(10.1)	2611	(2.5)	7818	(7.6)	
≧High school	322,493	(100.0)	258,656	(80.2)	44,267	(13.7)	17,525	(5.4)	26,742	(8.3)	19,570	(6.1)	4924	(1.5)	14,646	(4.5)	
**BMI**																	< 0.001
< 18.5	37,078	(100.0)	32,472	(87.6)	2928	(7.9)	1146	(3.1)	1782	(4.8)	1678	(4.5)	353	(1.0)	1325	(3.6)	
23–30	376,511	(100.0)	294,293	(78.2)	55,012	(14.6)	22,436	(6.0)	32,576	(8.7)	27,206	(7.2)	6855	(1.8)	20,351	(5.4)	
≧ 30	16,427	(100.0)	12,502	(76.1)	2369	(14.4)	925	(5.6)	1444	(8.8)	1556	(9.5)	450	(2.7)	1106	(6.7)	
**Smoking status**																	< 0.001
Never smoker	299,544	(100.0)	267,379	(89.3)	24,507	(8.2)					7658	(2.6)					
Smoker	125,429	(100.0)	66,845	(53.3)	35,802	(28.5)					22,782	(18.2)					
**Physical activity**																	< 0.001
Inactive	223,163	(100.0)	180,231	(80.8)	26,582	(11.9)	9902	(4.4)	16,680	(7.5)	16,350	(7.3)	3,621	(1.6)	12,729	(5.7)	
Low active	97,776	(100.0)	77,619	(79.4)	14,610	(14.9)	6227	(6.4)	8383	(8.6)	5547	(5.7)	1394	(1.4)	4153	(4.2)	
Fully active	105,269	(100.0)	78,568	(74.6)	18,492	(17.6)	8110	(7.7)	10,382	(9.9)	8209	(7.8)	2539	(2.4)	5670	(5.4)	
**Anemia**																	< 0.001
No	398,827	(100.0)	311,974	(78.2)	57,919	(14.5)	23,131	(5.8)	34,788	(8.7)	28,934	(7.3)	7149	(1.8)	21,785	(5.5)	
Yes	31,189	(100.0)	27,293	(87.5)	2390	(7.7)	1376	(4.4)	1014	(3.3)	1506	(4.8)	509	(1.6)	997	(3.2)	
**Systolic blood pressure**																	< 0.001
< 140	370,937	(100.0)	295,215	(79.6)	51,478	(13.9)	20,716	(5.6)	30,762	(8.3)	24,244	(6.5)	5872	(1.6)	18,372	(5.0)	
≧ 140	59,079	(100.0)	44,052	(74.6)	8831	(14.9)	3791	(6.4)	5040	(8.5)	6196	(10.5)	1786	(3.0)	4410	(7.5)	
**Fasting glucose (mg/dL)**																	< 0.001
< 126	412,318	(100.0)	326,513	(79.2)	57,621	(14.0)	23,517	(5.7)	34,104	(8.3)	28,184	(6.8)	7042	(1.7)	21,142	(5.1)	
≧ 126	17,698	(100.0)	12,754	(72.1)	2688	(15.2)	990	(5.6)	1698	(9.6)	2256	(12.7)	616	(3.5)	1640	(9.3)	
**Total cholesterol (mg/dL)**																	< 0.001
< 150	42,115	(100.0)	34,807	(82.6)	4684	(11.1)	1733	(4.1)	2951	(7.0)	2624	(6.2)	617	(1.5)	2007	(4.8)	
> 150	387,640	(100.0)	304,265	(78.5)	55,586	(14.3)	22,756	(5.9)	32,830	(8.5)	27,789	(7.2)	7035	(1.8)	20,754	(5.4)	
**High-density lipoprotein**																	< 0.001
< 35	358,698	(100.0)	285,983	(79.7)	48,592	(13.5)	20,879	(5.8)	27,713	(7.7)	24,123	(6.7)	6416	(1.8)	17,707	(4.9)	
≧ 35	41,594	(100.0)	28,629	(68.8)	8529	(20.5)	2552	(6.1)	5977	(14.4)	4436	(10.7)	844	(2.0)	3592	(8.6)	
**Low-density lipoprotein**																	< 0.001
< 160	353,523	(100.0)	279,473	(79.1)	49,359	(14.0)	20,216	(5.7)	29,143	(8.2)	24,691	(7.0)	6246	(1.8)	18,445	(5.2)	
≧160	44,237	(100.0)	33,463	(75.6)	7312	(16.5)	3061	(6.9)	4251	(9.6)	3462	(7.8)	962	(2.2)	2500	(5.7)	
**Triglycerides**																	< 0.001
< 200	383,834	(100.0)	308,846	(80.5)	51,407	(13.4)	21,963	(5.7)	29,444	(7.7)	23,581	(6.1)	6391	(1.7)	17,190	(4.5)	
≧ 200	45,934	(100.0)	30,230	(65.8)	8867	(19.3)	2531	(5.5)	6336	(13.8)	6837	(14.9)	1261	(2.7)	5576	(12.1)	
**Proteinuria**																	< 0.001
Normal	381,477	(100.0)	300,447	(78.8)	54,610	(14.3)	22,299	(5.8)	32,311	(8.5)	26,420	(6.9)	6732	(1.8)	19,688	(5.2)	
Minimal proteinuria	26,891	(100.0)	19,494	(72.5)	4250	(15.8)	1360	(5.1)	2890	(10.7)	3,147	(11.7)	638	(2.4)	2509	(9.3)	
Overt proteinuria	2394	(100.0)	1747	(73.0)	299	(12.5)	103	(4.3)	196	(8.2)	348	(14.5)	84	(3.5)	264	(11.0)	
**Uric acid level**																	< 0.001
< 7	318,589	(100.0)	263,077	(82.6)	37,974	(11.9)	16,737	(5.3)	21,237	(6.7)	17,538	(5.5)	4688	(1.5)	12,850	(4.0)	
≧ 7	111,427	(100.0)	76,190	(68.4)	22,335	(20.0)	7770	(7.0)	14,565	(13.1)	12,902	(11.6)	2970	(2.7)	9932	(8.9)	
**C-reactive protein (mg/L)**																	< 0.001
< 1	294,833	(100.0)	234,540	(79.6)	41,314	(14.0)	17,235	(5.8)	24,079	(8.2)	18,979	(6.4)	4899	(1.7)	14,080	(4.8)	
1–2.9	77,514	(100.0)	59,799	(77.1)	11,182	(14.4)	4303	(5.6)	6879	(8.9)	6533	(8.4)	1606	(2.1)	4927	(6.4)	
3–9.9	34,288	(100.0)	26,111	(76.2)	4931	(14.4)	1800	(5.2)	3131	(9.1)	3246	(9.5)	757	(2.2)	2489	(7.3)	
≧ 10	11,697	(100.0)	8628	(73.8)	1777	(15.2)	680	(5.8)	1097	(9.4)	1292	(11.0)	275	(2.4)	1017	(8.7)	

^§^Regular Drinker include ex-drinker group.

After adjusted 15 confounders, modest drinker had an 8% decrease in mortality risk (hazard ratio, HR 0.92; 95% CI 0.86 to 0.97), 34% decrease in diabetes mellitus mortality risk, 14% decrease in expanded CVD mortality risk and 26% decrease in respiratory system mortality risk compared to non-drinker (Table 2).

**Table 2:** Mortality risks by alcohol drinking status, compared to non-drinker.

	ICD 9	Total deaths	Non-drinker (n = 339,267)		Modest drinker (n = 64,129)			Regular drinker^§^ (n = 30,915)		
			Deaths	HRs	Deaths	HRs	95% CI	Deaths	HRs	95% CI
**All cause**	001–998	11,031	7156	1.00	1947	0.92^#^	(0.86, 0.97)	1928	1.43*	(1.35, 1.52)
Male		6931	3453	1.00	1689	0.92^#^	(0.86, 0.98)	1789	1.45*	(1.37, 1.55)
Female		4100	3703	1.00	258	0.92	(0.80, 1.07)	139	1.39*	(1.15, 1.68)
**All cancer**	140–208	4472	2876	1.00	831	0.97	(0.89, 1.06)	765	1.52*	(1.39, 1.66)
Male		2772	1333	1.00	723	0.99	(0.90, 1.09)	716	1.56*	(1.41, 1.72)
Female		1700	1543	1.00	108	0.88	(0.70, 1.10)	49	1.27	(0.92, 1.75)
Oral cancer	140–149	119	32	1.00	35	2.35*	(1.38, 4.01)	52	4.65*	(2.79, 7.77)
Oesophagus	150	98	15	1.00	26	3.83*	(1.90, 7.73)	57	12.14*	(6.32, 23.33)
Stomach	151	285	196	1.00	50	0.90	(0.63, 1.27)	39	1.20	(0.82, 1.78)
Colon rectum	153–154	458	318	1.00	85	0.98	(0.74, 1.30)	55	1.17	(0.85, 1.61)
Liver	155	961	545	1.00	192	1.07	(0.89, 1.29)	224	2.00*	(1.67, 2.40)
Pancreas	157	184	123	1.00	36	1.23	(0.80, 1.90)	25	1.52	(0.92, 2.52)
Lung	162	936	587	1.00	181	0.85	(0.70, 1.03)	168	1.36*	(1.12, 1.65)
Breast	174	179	166	1.00	9	0.67	(0.31, 1.44)	4	1.34	(0.48, 3.76)
Prostate	185	104	51	1.00	31	1.22	(0.76, 1.97)	22	1.46	(0.85, 2.51)
Bladder	188	63	38	1.00	17	1.13	(0.58, 2.20)	8	0.80	(0.34, 1.89)
Diabetes mellitus	250	672	498	1.00	85	0.66^#^	(0.50, 0.86)	89	0.81	(0.62, 1.07)
**Cardiovascular disease**	390–459	2115	1376	1.00	370	0.93	(0.81, 1.06)	369	1.39*	(1.22, 1.59)
Ischemic heart disease	410–414	594	374	1.00	110	0.94	(0.74, 1.20)	110	1.37*	(1.07, 1.75)
Stroke	430–438	874	571	1.00	148	0.91	(0.74, 1.12)	155	1.41*	(1.14, 1.73)
Expanded CVD		3064	2073	1.00	494	0.86^#^	(0.77, 0.96)	497	0.20	(1.07, 1.35)
**Respiratory system**	460–519	645	420	1.00	106	0.74^#^	(0.58, 0.94)	119	1.24	(0.98, 1.56)
COPD	491–496	256	160	1.00	43	0.68^#^	(0.47, 1.00)	53	1.16	(0.81, 1.66)
**Digestive system**	520–579	731	440	1.00	111	0.99	(0.78, 1.26)	180	2.41*	(1.95, 2.99)
Cirrhosis	571	395	227	1.00	59	0.99	(0.71, 1.38)	109	2.90*	(2.18, 3.84)
Kidney disease	580–589	277	199	1.00	39	0.78	(0.52, 1.16)	39	0.85	(0.56, 1.30)
Accidents	800–949	681	396	1.00	151	1.16	(0.93, 1.43)	134	1.80*	(1.44, 2.25)
Suicide	950–959	299	202	1.00	44	0.65^#^	(0.44, 0.95)	53	1.51*	(1.05, 2.15)

Hazard ratios are adjusted for age, gender, education, body mass index, smoking, physical activity, systolic blood pressure, fasting blood glucose, anemia, total cholesterol, high-density lipoprotein cholesterol, low-density lipoprotein cholesterol, triglycerides, proteinuria, uric acid level and C-reactive protein in a multivariate Cox model when appropriate.

*Expanded CVD* CVD plus diabetes plus kidney disease.

^#^Indicates that the statistically significant reduction in HRs.

*Indicates a statistically significant increase in HR.

^§^Regular Drinker include ex-drinker group.

As the majority of drinkers (64.6% in total and 72.2% in males) also smoked, further analysis of non-smoking drinking HRs were needed to avoid the mixing effect of smoking and drinking. Besides most smokers were male in this study cohort. Table 3 shows the comparison of mortality risks of males by drinking status by different causes of deaths, with non-drinkers as the reference. After controlled for 14 confounders, regular drinkers who were nonsmokers had significant increase mortality risk in all cause (HR 1.34; 95% CI 1.19 to 1.50), all cancer (HR 1.39; 95% CI 1.17 to 1.66), oesophagus cancer (HR 9.21; 95% CI 3.48 to 24.39), liver cancer (HR 2.06; 95% CI 1.50 to 2.82) and accidents (HR 1.57; 95% CI 1.02 to 2.43). However, modest drinkers who were non-smokers had significantly 19% lower mortality risk compared to the non-drinker (HR 0.81; 95% CI 0.74 to 0.89), especially in all cancer (HR 0.84; 95% CI 0.73 to 0.97), lung cancer (HR 0.58; 95% CI 0.40 to 0.83), diabetes (HR 0.55; 95% CI 0.34 to 0.88), expanded CVD (HR 0.81; 95% CI 0.67 to 0.97) and respiratory system disease (HR 0.54; 95% CI 0.33 to 0.86) when compared to the non-drinker.

**Table 3:** Mortality risks by alcohol drinking and smoking status, compared to non-drinker.

	Total deaths	Non-drinker		Modest drinker						Regular drinker^§^					
				Never smoker			Smoker			Never smoker			Smoker		
		Deaths	HRs	Deaths	HRs	95% CI	Deaths	HRs	95% CI	Deaths	HRs	95% CI	Deaths	HRs	95% CI
All cause	10,862	7156	1.00	513	0.81^#^	(0.74, 0.89)	1292	1.11*	(1.04, 1.19)	348	1.34	(1.19, 1.50)	1553	1.70*	(1.60, 1.81)
**All cancer**	4413	2876	1.00	218	0.84^#^	(0.73, 0.97)	565	1.23*	(1.11, 1.35)	140	1.39	(1.17, 1.66)	614	1.86*	(1.69, 2.05)
Oral cancer	117	32	1.00	5	1.21	(0.42, 3.48)	29	3.58*	(2.07, 6.20)	6	2.93	(1.02, 8.45)	45	6.70*	(4.02, 11.19)
Oesophagus	98	15	1.00	5	3.16*	(1.13, 8.84)	21	5.50*	(2.62, 11.53	7	9.21	(3.48, 24.39	50	17.01*	(8.83, 32.77)
Stomach	283	196	1.00	16	0.95	(0.57, 1.59)	33	0.97	(0.64, 1.46)	6	0.77	(0.32, 1.88)	32	1.48	(0.99, 2.22)
Colon rectum	452	318	1.00	28	1.00	(0.66, 1.51)	52	1.08	(0.78, 1.50)	12	1.11	(0.61, 2.04)	42	1.32	(0.94, 1.86)
Liver	945	545	1.00	47	0.87	(0.64, 1.19)	132	1.32*	(1.07, 1.62)	44	2.06	(1.50, 2.82)	177	2.25*	(1.86, 2.72)
Pancreas	181	123	1.00	10	1.02	(0.53, 1.96)	24	1.40	(0.85, 2.29)	6	1.62	(0.70, 3.72)	18	1.48	(0.85, 2.58)
Lung	923	587	1.00	31	0.58^#^	(0.40, 0.83)	139	1.30*	(1.06, 1.60)	26	1.19	(0.79, 1.79)	140	1.92*	(1.57, 2.36)
Prostate	104	51	1.00	8	1.16	(0.55, 2.47)	23	1.30	(0.78, 2.17)	3	1.02	(0.32, 3.29)	19	1.61	(0.93, 2.81)
Bladder	62	38	1.00	4	0.90	(0.27, 2.96)	12	1.49	(0.73, 3.06)	2	0.66	(0.09, 4.90)	6	1.04	(0.42, 2.56)
Diabetes mellitus	662	498	1.00	19	0.55^#^	(0.34, 0.88)	56	0.84	(0.62, 1.13)	12	0.68	(0.38, 1.21)	77	1.00	(0.76, 1.33)
**Cardiovascular disease**	2085	1376	1.00	94	0.82	(0.66, 1.02)	250	1.12	(0.96, 1.30)	69	1.30	(1.00, 1.68)	296	1.63*	(1.42, 1.88)
Ischemic heart disease	585	374	1.00	21	0.66	(0.42, 1.03)	81	1.24	(0.95, 1.62)	26	1.75	(1.15, 2.66)	83	1.54*	(1.18, 2.00)
Stroke	861	571	1.00	32	0.69^#^	(0.47, 1.00)	105	1.19	(0.95, 1.50)	28	1.32	(0.88, 1.98)	125	1.69*	(1.36, 2.11)
Expanded CVD	3018	2073	1.00	132	0.81^#^	(0.67, 0.97)	322	1.00	(0.88, 1.14)	90	1.14	(0.91, 1.43)	401	1.39*	(1.23, 1.57)
**Respiratory system**	632	420	1.00	19	0.54^#^	(0.33, 0.86)	78	0.94	(0.73, 1.21)	20	1.19	(0.73, 1.94)	95	1.49*	(1.17, 1.90)
COPD	247	160	1.00	4	0.33^#^	(0.12, 0.88)	33	1.00	(0.67, 1.50)	7	0.94	(0.38, 2.30)	43	1.63*	(1.13, 2.37)
**Digestive system**	718	440	1.00	37	0.98	(0.68, 1.41)	64	1.08	(0.81, 1.43)	34	2.25	(1.56, 3.24)	143	2.63*	(2.10, 3.28)
Cirrhosis	386	227	1.00	18	0.96	(0.58, 1.58)	34	1.05	(0.71, 1.55)	20	2.46	(1.52, 3.97)	87	3.07*	(2.29, 4.11)
Kidney disease	271	199	1.00	19	1.31	(0.80, 2.14)	16	0.51^#^	(0.29, 0.90)	9	1.24	(0.60, 2.54)	28	0.76	(0.48, 1.21)
Accidents	670	396	1.00	42	1.00	(0.71, 1.41)	100	1.33*	(1.05, 1.69)	23	1.57	(1.02, 2.43)	109	2.01*	(1.59, 2.53)
Suicide	294	202	1.00	15	0.74	(0.42, 1.31)	24	0.81	(0.52, 1.28)	5	0.93	(0.38, 2.27)	48	2.27*	(1.58, 3.26)

Hazard ratios are adjusted for age, education, body mass index, physical activity, systolic blood pressure, fasting blood glucose, anemia, total cholesterol, high-density lipoprotein cholesterol, low-density lipoprotein cholesterol, triglycerides, proteinuria, uric acid level and C-reactive protein in a multivariate Cox model when appropriate.

*Expanded CVD* CVD plus diabetes plus kidney disease.

^#^Indicates that the statistically significant reduction in HRs.

*Indicates a statistically significant increase in HR.

^§^Regular Drinker include ex-drinker group.

**Figure 1:**
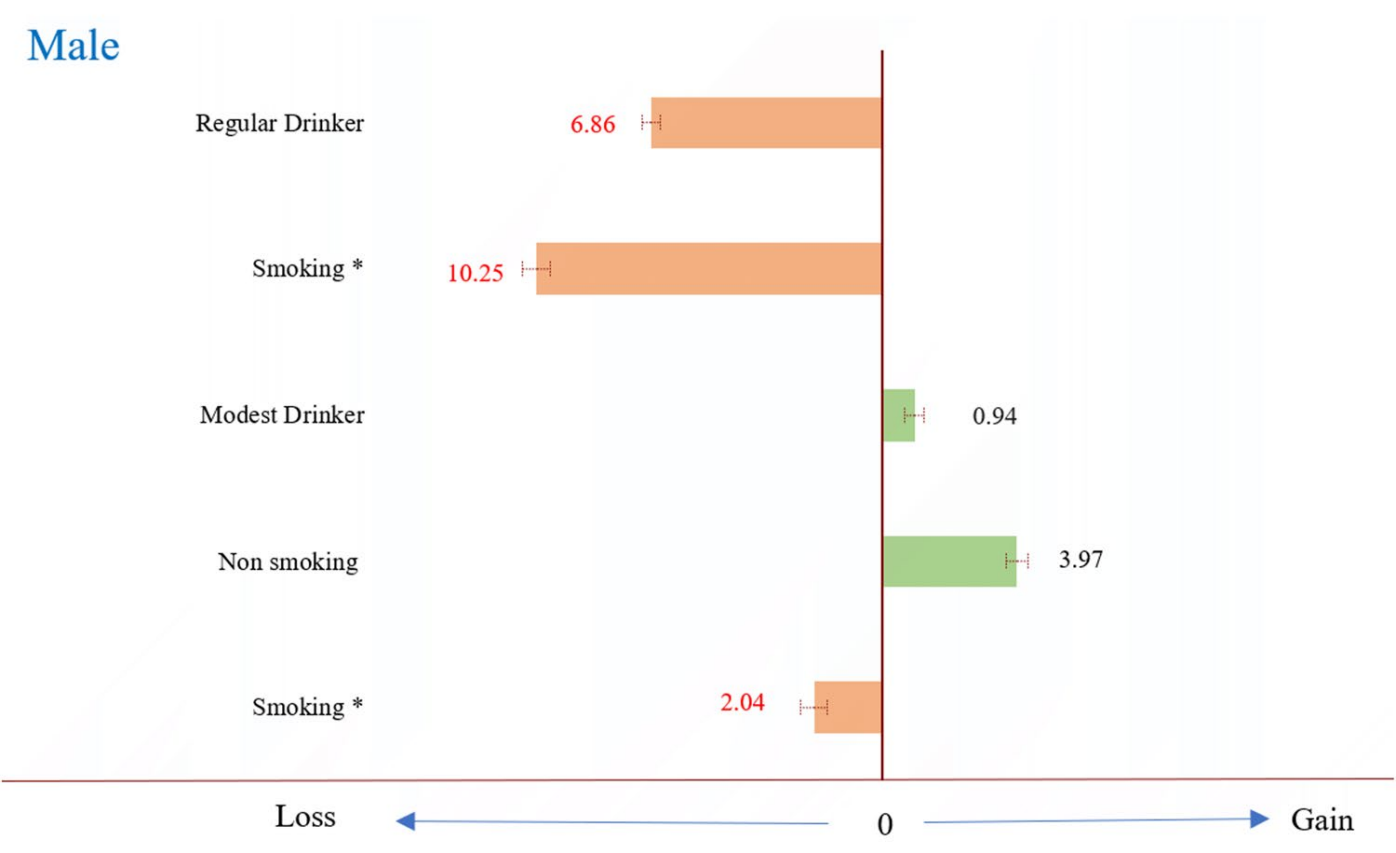
The difference in life expectancy among drinking status, compared to non-drinker. *Reference group is nonsmoking non-drinker. x-axis means the difference in life expectancy (years) among drinking status, compared to non-drinker. Regular Drinker include ex-drinker group.

As shown in Table 1, the alcohol consumption rate is quite different between males and females. The rate of modest drinking and regular drinking in males is 22.6% and 12.1%, whereas 5.8% and 2.3 in females. Since the number of female deaths is relatively small, we only analyze the life expectancy in males. Compared with nondrinkers, regular drinkers in males shortened life by 6.86 years (95% CI 6.58–7.14 years), while regular drinkers with smoking loss 10.25 years (95% CI 9.84–10.66 years) when compared to nonsmoking non-drinker. In the other hand, male modest drinkers gain 0.94 years (95% CI 0.65–1.23 years) and male modest drinkers who were never smokers gain 3.97 years (95% CI 3.65–4.29 years), but loss 2.04 years (95% CI 1.64–2.44 years) if smoking (Fig. 1).

## Discussion

In this cohort study of 430,270 adults in Taiwan, the results suggested that modest drinking is associated with significantly reduced risk of all-cause mortality, diabetes mellitus, expanded CVD (diabetes, cardiovascular disease, and chronic kidney disease), respiratory system disease (include COPD), and suicide; whereas a 2 to 4 folds increased risk of oral cancer and esophageal cancer. The results also showed that male modest drinkers gain 0.94 years (95% CI 0.65–1.23 years) and male modest drinkers who were never smokers gain 3.97 years (95% CI 3.65–4.29 years) compared to non-drinker. It appears that a little drinking could be better than none. However, drinking beyond modest amount led to a large loss of life expectancy of 7–10 years in males. Given that drinkers are prone to cross the line from limited benefits to grave consequences, clinicians should cautious in their recommendations.

This large population-based cohort study is the first study to use the life expectancy to assess the absolute risk from the different levels of alcohol consumption. The mortality risks of various diseases in males were analyzed from the data of the National Cancer Registry and National Death File. On the other hand, the co-use of smoking in drinkers is common^23,24^. To avoid the mixing effect of smoking and drinking, the subgroup analysis was conducted to make the results closer to real world because of the high co-use rate, 72.2% of males in our study. Moreover, in order to clarify the previously proposed methodological problems of abstainers’ misclassification, sick quitter hypothesis, and potentially confounding factors^17^, we categorized drinking status of ex-drinkers into regular drinker to avoid misclassifying the former drinkers as abstainers and controlled 15 confounders to minimize confounding. There were still some limitations in this study. First, we collected the self-reported responses at baseline, but the participants might change their consumption behavior during follow-up. Recall bias may also exist in these self-report materials. Changes in drinking behavior, whether increasing or decreasing, and underreporting of alcohol use may affect the quality of drinking data used in this study. All subjects in this study aged 20 years or older were recruited by the MJ Health Group, Taiwan, to participate in a standard health-screening program between 1994 and 2008. A total of 11,031 deaths were identified with a median follow-up period of 8.8 years. According to the previous study^25^, women were more likely than men to be former rather than current drinkers in 87 of 96 age-specific comparisons, and in the majority of age groups in 30 of the 32 countries, meaning that men are less likely to quit alcohol than women. Take Japan as an example, the rate of former drinker in men was only 3.5%. The period of our study is from 1994 to 2008, and the average follow-up period was 8.8 years. According to previous literature, the rate of drinkers who quit alcohol is not high, we speculate that the impact on the results should be limited. However, the inability to continuously track drinking status is indeed one of the main limitations of this study, and we must make improvements in future study design. Secondly, there might be residual confounding factors in addition to the 15 confounders we controlled, such as the mental and socioeconomic status in suicide analysis. Lastly, the case numbers in certain death categories were too small, such as breast and bladder cancer, and may affect the statistical accuracy.

This study of Asians is similar to previous studies of Asian alcohol consumption, in that the effects of alcohol on Asians cannot be underestimated. The systematic analysis for the Global Burden of Disease Study 2016^26^ indicated that the prevalence of abstainers in Taiwan was 82% in females and 56% in males in 2016, and was 84% in females and 61% in males in 1990. The prevalence of abstainers in our study was 91.9% in females and 65.4% in males, higher than the global statistics. Such a difference has no obvious explanation. Underreporting of alcohol consumption may lead the risks to be biased toward the null hypothesis^18^. The alcohol-attributable fraction (AF) of all cancers was highest in the Western Pacific region around the world, with 7.1% for incidence and 7.8% for mortality, which was higher than the global rates of 5.5% and 5.8% in 2012^26^. However, the opposite research indicated that modest drinking may be beneficial to health within Asia population^27–29^. Although there is no global consensus on alcohol consumption recommendation, countries usually have their own standards. According to the Dietary Guidelines for Americans 2015–2020, women can drink up to 14 g of pure alcohol per day and men can drink 28 g per day. Previous research had also indicated that the light to modest drinking could decrease the all-cause mortality^4,30^. However, the Korean meta-analysis proposed that mild alcohol consumption have no benefit for all-cause, cancer-related, and cardiovascular mortality^31^. Zaitsu et al. indicated that the light to modest drinking elevated the all-cancer incidence^32^. Another Japanese pooled analysis revealed an increased dose–response relationship between alcohol and colorectal cancer incidence, and the relation was more apparent in Japanese than in Western populations^33^. It’s well known that East Asians had higher proportion of genetic variant of the aldehyde dehydrogenase 2 (ALDH2) enzyme, identified as ALDH2*2, which cause lower metabolic efficiency of reactive aldehyde carcinogens^34^. These results of previous studies showed that the harm of alcohol might be greater in Asia. Our study shows only 0.94 years of life gain in modest drinking group, and the benefits will be eliminated to 1.49 year life loss if smoking. About two thirds of modest drinkers smoke, meaning that only a small percentage of people can benefit from modest drinking. Moreover, alcohol is addictive and affects judgment, making it difficult to control the amount within two drinks. Once more than two drinks each time and three times a week, the life expectancy in males may reduced by 6.86 years compared with nondrinkers in this study.

This study aims to examine the effect on different diseases of modest dirking. The CVD benefit of modest drinking are the most frequently mentioned. Previous studies have strongly indicated that modest drinking was beneficial to CVD^13–15,35,36^. The possible mechanisms might be the biologic and molecular pathways include enhanced insulin sensitivity, increased high-density lipoprotein (HDL) cholesterol, and improved endothelial function by decreased tissue injury via limited ischemia/reperfusion ratio induced by ethanol^35,37^. In contrast, Holmes et al. indicated that there were no protective effects toward CVD in low-moderate alcohol consumption group based on the Mendelian randomization analysis^38^. Our study also reveals non-significant CVD risk between modest-drinker and non-drinker (Table 2). The effect of modest drinking on CVD is still controversial. However, in terms of expanded CVD (i.e., CVD plus type 2 diabetes and kidney disease), the risk is significantly reduced by 14% compared to non-drinkers. Consistent with our results, other studies also revealed the reduced type 2 diabetes risk among modest drinkers in both Western and Asian populations by improving the insulin sensitivity^7,9,29,39^.

Our study proposed that the modest-drinking group has no significant effects on cancer mortality, both generally or in any particular cancer. The effects on the different types of alcohol related cancers in previous research were still controversial^32,33,40–43^, but these studies included few Asian populations or did not adjust the interaction of smoking and other confounding factors. Similar to the results of life expectancy, the all-cancer mortality risk is decreased only in modest drinkers who don’t smoke in our subgroup analysis by smoking status, and only significant in lung cancer when viewed individually. Previous research had similar findings of lung cancer^8,44^. The possible protective effects might be due to anti-carcinogenic and chemo-preventive properties of the flavonoids^45,46^. On the other hand, our results discover that even modest drinking significantly increased 3.83-folds mortality risk of esophagus cancer and 2.35-folds risk of oral cancer. The study using the most recent International Agency for Research on Cancer (IARC) monograph also showed that the cancers with the highest PAFs were cancers of oesophagus, pharynx, and lip and oral cavity^47^. The risks are much higher than 1.17 times of oropharyngeal cancer and 1.30 times of esophageal cancer in previous meta-analysis^43^. The potential cause might be the high prevalence of ALDH2*2 allele in East Asians, and the evidence of the carcinogenic effect is strong in esophageal cancer and head and neck cancer^48^. In addition, the concurrent use of tobacco and alcohol is common and increasing over time, especially among socioeconomically disadvantaged population^24,49^. The mortality risk of esophageal cancer is more than 5 times in modest drinkers who smoke compared to non-drinker. This is an important issue about cancer prevention we must pay attention to.

In conclusions, modest drinkers, no more than one drink a day, had benefits and could gain nearly 1 year in life expectancy, in contrast to a loss of nearly 7 years if drinking more than that. The loss exceeded 10 years if drinkers also smoked, as did majority (65–80%) of drinkers. Given the reality that drinkers are prone to cross the line, clinicians should balance the risks and benefits of drinking, as well as the understanding of whether the patient is at risk for addiction.

## Methods

### Ethics

The study was approved by the Institutional Review Boards at the China Medical University (CRREC-107-092) and National Health Research Institutes (EC0981201-E), Taiwan. The requirement for written informed consent was waived by the Institutional Review Board. All analyses adhered to the guidelines and regulations of the ethics committee of National Health Research Institutes.

### Data collection

All subjects aged 20 years or older were recruited by the MJ Health Group, Taiwan, to participate in a standard health-screening program between 1994 and 2008. Excluding individuals without drinking data, a study cohort of 430,270 individuals was established. Participants had tests for blood, urine, body measurements, functional tests, physical examination and completed a comprehensive health history questionnaire to collect medical history and epidemiological data. Drinking status was defined from questionnaire according to not only self-report never or occasional drinker, ex-drinker and drink alcohol three times or more a week and two or more drinks a week, the amount of alcohol each time was estimated by number of drinks. Drinking status was categorized into four levels: “non-drinker”, “ex-drinker”, “modest drinker” (drinking alcohol less three times a week and less two drinks each time) and “regular drinker” (drinking alcohol three times or more a week and two or more drinks each time). Smoking was defined according to answer “Do you now smoke cigarettes?” and categorized into 2 groups: “never smokers” and “smokers”. Considering the portion of ex-drinkers and ex-smokers were very small (3% and 6% respectively) but hazard ratios (HRs) were large and were comparable with regular drinkers and current smokers in this study cohort. In additions, most ex-drinkers and ex-smokers might quit because alcohol and cigarettes had led some health problems. That was why we combined ex-drinker and ex-smokers into “regular drinker” and “current smoker”. Leisure time physical activity (LTPA) volume was ascertained by three multiple choice questions. The duration and intensity of weekly physical activity were converted into MET (Metabolic Equivalent for Task)-hour/week. LTPA = METs (metabolic equivalents) × time (h) and was classified into three levels: “inactive” (< 3.75 MET-h/week), “low active” (exercised 15–29 min a day on average, or 3.75–7.49 MET-h/week) and “fully active”, with ≧ 7.5 MET-hour/week or ≧ 30 min/day, met the current exercise recommendation of ≧ 150 min/week. Causes of death were classified according to International Classification of Diseases, 9th version (ICD-9). Health outcome variables were all causes mortality (ICD-9 codes 001–998), all cancer (ICD-9 codes 140–208), oral cancer (ICD-9 codes 140–149), esophageal cancer (ICD-9 codes 150), stomach cancer (ICD-9 codes 151), colon rectum cancer (ICD-9 codes 153–154), liver cancer (ICD-9 codes 155), pancreas cancer (ICD-9 codes 157), lung cancer (ICD-9 codes 162), breast cancer (ICD-9 codes 174), prostate cancer (ICD-9 codes 185), bladder cancer (ICD-9 codes 188), Diabetes mellitus (ICD-9 codes 250), cardiovascular disease (CVD) (ICD-9 codes 390–459), ischemic heart disease (ICD-9 codes 410–414), stroke (ICD-9 codes 430–438), respiratory system disease (ICD-9 codes 460–519), COPD (ICD-9 codes 491–496), digestive system disease (ICD-9 codes 520–579), cirrhosis (ICD-9 codes 571), kidney disease (ICD-9 codes 580–589), accidents (ICD-9 codes 800–949) and suicide (ICD-9 codes 950–959). Expanded CVD was CVD plus diabetes plus kidney disease.

### Follow-up

Using the unique national identification numbers, subjects were each matched with the National Cancer Registry and National Death File between 1997 and 2008. A total of 11,031 deaths were identified with a median follow-up period of 8.8 years. Informed consent was obtained to authorize the processing and analyzing of the data. The study was approved by the Institutional Review Boards at the National Health Research Institutes, Taiwan. A detailed description of this method has been reported earlier.

### Statistical analysis

Risk predictors were subjected to Cox proportional hazards regression analysis to identify significant predictors in multivariate models. Hazard ratios (HRs) and 95% confidence intervals (CIs) were estimated for each variable. HRs were calculated using Cox proportional hazards model, with adjustments made, when appropriate, for confounders. We selected confounders based on literature that address the increase risk for drinking, with most of them similar to confounders we used in other studies^50,51^. HRs were adjusted for 15 confounding variables, including 2 continuous and 13 categorical variables. The continuous confounding variables were age, and C-reactive protein level; the categorical variables were gender, education (Middle school or below, High school, Junior college, College or above), BMI (< 18.5, 18.5–23, 23–25, 23–30, ≧ 30), smoking (nonsmoker, ex-smoker, current smoker), physical activity (inactive, somewhat active, fully active), anemia, fasting glucose (< 126 mg/dL, ≧ 126 mg/dL), Systolic blood pressure (< 140 mmHg, ≧ 140 mmHg), total cholesterol (< 150 mg/dL, ≧ 150 mg/dL), High-density lipoprotein (< 35 mg/dL, ≧ 35 mg/dL), Low-density lipoprotein (< 160 mg/dL, ≧ 160 mg/dL), triglycerides (< 200 mg/dL, ≧ 200 mg/dL), proteinuria (normal, minimal proteinuria, overt proteinuria), and uric acid level (< 7 mg/dL, ≧ 7 mg/dL). Life expectancy was calculated based on Chiang^52^. LE1 for drinkers and LE2 for nondrinkers. LEV2-LEV1 = lost years of life. After adjusted for age and gender, we found the odds ratio between smoking and drinking is much larger than odds ratios between BMI and drinking or physical activity and drinking. Therefore, we performed further analysis of mortality risks by smoking status rather than other important lifestyle risk factors of health-related outcomes such as BMI or physical activity. All statistical tests were two-sided with the alpha level set at 0.05 and all statistical analyses were performed with SAS 9.4 (SAS Institute Inc., Cary, NC).

## Supplementary Information

Supplementary Tables.
