# Supplementary material for: The effects of modest drinking on life expectancy and mortality risks: a population-based cohort study
Source: Sci Rep. 2022 May 6;12:7476. doi: 10.1038/s41598-022-11427-x (PMC9076667; doi:10.1038/s41598-022-11427-x)
Supplement: Supplementary file 1 — Supplementary Tables. [file 41598_2022_11427_MOESM1_ESM.docx]

**Table S1. Demographics and clinical characteristics by drinking status**

|  | **Total** | |  | **Non-Drinker** | |  | **Modest Drinker** | | | | | | | |  |  | **Ex-Drinker** | |  | **Regular Drinker** | | | | | | | |
| --- | --- | --- | --- | --- | --- | --- | --- | --- | --- | --- | --- | --- | --- | --- | --- | --- | --- | --- | --- | --- | --- | --- | --- | --- | --- | --- | --- |
|  |  |  |  |  |  |  |  | |  | Never smoker | |  | Smoker | |  |  |  |  |  |  | |  | Never smoker | |  | Smoker | |
|  | n | (%) |  | n | (%) |  | n | (%) |  | n | (%) |  | n | (%) |  |  | n | (%) |  | n | (%) |  | n | (%) |  | n | (%) |
| **Total** | 430,270 | (100.0) |  | 339,267 | (78.9) |  | 60,309 | (14.0) |  | 24,507 | (5.7) |  | 35,802 | (8.3) |  |  | 13,146 | (3.1) |  | 17,548 | (4.1) |  | 3,307 | (0.8) |  | 14,241 | (3.3) |
| **Age** |  |  |  |  |  |  |  |  |  |  |  |  |  |  |  |  |  |  |  |  |  |  |  |  |  |  |  |
| 20-39 | 243,792 | (100.0) |  | 199,709 | (81.9) |  | 30,478 | (12.5) |  | 11,382 | (4.7) |  | 19096 | (7.8) |  |  | 5,134 | (2.1) |  | 8,471 | (3.5) |  | 1,273 | (0.5) |  | 7,198 | (3.0) |
| 40-64 | 158,361 | (100.0) |  | 118,394 | (74.8) |  | 25,728 | (16.2) |  | 11,659 | (7.4) |  | 14069 | (8.9) |  |  | 5,932 | (3.7) |  | 8,307 | (5.2) |  | 1,875 | (1.2) |  | 6,432 | (4.1) |
| ≧65 | 28,117 | (100.0) |  | 21,164 | (75.3) |  | 4,103 | (14.6) |  | 1,466 | (5.2) |  | 2637 | (9.4) |  |  | 2,080 | (7.4) |  | 770 | (2.7) |  | 159 | (0.6) |  | 611 | (2.2) |
| **Gender** |  |  |  |  |  |  |  |  |  |  |  |  |  |  |  |  |  |  |  |  |  |  |  |  |  |  |  |
| Male | 210,738 | (100.0) |  | 137,664 | (65.3) |  | 47,540 | (22.6) |  | 14,999 | (7.1) |  | 32541 | (15.4) |  |  | 9,995 | (4.7) |  | 15,539 | (7.4) |  | 2,519 | (1.2) |  | 13,020 | (6.2) |
| Female | 219,532 | (100.0) |  | 201,603 | (91.8) |  | 12,769 | (5.8) |  | 9,508 | (4.3) |  | 3261 | (1.5) |  |  | 3,151 | (1.4) |  | 2,009 | (0.9) |  | 788 | (0.4) |  | 1221 | (0.6) |
| **Education** |  |  |  |  |  |  |  |  |  |  |  |  |  |  |  |  |  |  |  |  |  |  |  |  |  |  |  |
| ≦Middle school | 103,096 | (100.0) |  | 77,218 | (74.9) |  | 15,328 | (14.9) |  | 6,668 | (6.5) |  | 8660 | (8.4) |  |  | 4,703 | (4.6) |  | 5,847 | (5.7) |  | 1,157 | (1.1) |  | 4,690 | (4.5) |
| ≧High school | 322,619 | (100.0) |  | 258,656 | (80.2) |  | 44,267 | (13.7) |  | 17,525 | (5.4) |  | 26742 | (8.3) |  |  | 8,225 | (2.5) |  | 11,471 | (3.6) |  | 2,104 | (0.7) |  | 9,367 | (2.9) |
| **BMI** |  |  |  |  |  |  |  |  |  |  |  |  |  |  |  |  |  |  |  |  |  |  |  |  |  |  |  |
| <18.5 | 37,093 | (100.0) |  | 32,472 | (87.5) |  | 2,928 | (7.9) |  | 1,146 | (3.1) |  | 1782 | (4.8) |  |  | 865 | (2.3) |  | 828 | (2.2) |  | 110 | (0.3) |  | 718 | (1.9) |
| 23-30 | 376,732 | (100.0) |  | 294,293 | (78.1) |  | 55,012 | (14.6) |  | 22,436 | (6.0) |  | 32576 | (8.6) |  |  | 11,541 | (3.1) |  | 15,886 | (4.2) |  | 2,997 | (0.8) |  | 12,889 | (3.4) |
| ≧30 | 16,445 | (100.0) |  | 12,502 | (76.0) |  | 2,369 | (14.4) |  | 925 | (5.6) |  | 1444 | (8.8) |  |  | 740 | (4.5) |  | 834 | (5.1) |  | 200 | (1.2) |  | 634 | (3.9) |
| **Physical activity** |  |  |  |  |  |  |  |  |  |  |  |  |  |  |  |  |  |  |  |  |  |  |  |  |  |  |  |
| Inactive | 223,291 | (100.0) |  | 180,231 | (80.7) |  | 26,582 | (11.9) |  | 9,902 | (4.4) |  | 16680 | (7.5) |  |  | 6,803 | (3.0) |  | 9,675 | (4.3) |  | 1,620 | (0.7) |  | 8,055 | (3.6) |
| Low active | 97,815 | (100.0) |  | 77,619 | (79.4) |  | 14,610 | (14.9) |  | 6,227 | (6.4) |  | 8383 | (8.6) |  |  | 2,311 | (2.4) |  | 3,275 | (3.3) |  | 598 | (0.6) |  | 2,677 | (2.7) |
| Fully active | 105,341 | (100.0) |  | 78,568 | (74.6) |  | 18,492 | (17.6) |  | 8,110 | (7.7) |  | 10382 | (9.9) |  |  | 3,823 | (3.6) |  | 4,458 | (4.2) |  | 1,054 | (1.0) |  | 3,404 | (3.2) |
| **Smoking status** |  |  |  |  |  |  |  |  |  |  |  |  |  |  |  |  |  |  |  |  |  |  |  |  |  |  |  |
| Never smoker | 299,544 | (100.0) |  | 267379 | (89.3) |  | 24507 | (8.2) |  |  |  |  |  |  |  |  | 4351 | (1.5) |  | 3307 | (1.1) |  |  |  |  |  |  |
| Smoker | 125,429 | (100.0) |  | 66,845 | (53.3) |  | 35,802 | (28.5) |  |  |  |  |  |  |  |  | 8,541 | (6.8) |  | 14,241 | (11.4) |  |  |  |  |  |  |
| **Anemia** |  |  |  |  |  |  |  |  |  |  |  |  |  |  |  |  |  |  |  |  |  |  |  |  |  |  |  |
| No | 399,044 | (100.0) |  | 311974 | (78.2) |  | 57919 | (14.5) |  | 23131 | (5.8) |  | 34788 | (8.7) |  |  | 12168 | (3.0) |  | 16983 | (4.3) |  | 3161 | (0.8) |  | 13822 | (3.5) |
| Yes | 31,226 | (100.0) |  | 27,293 | (87.4) |  | 2,390 | (7.7) |  | 1,376 | (4.4) |  | 1014 | (3.2) |  |  | 978 | (3.1) |  | 565 | (1.8) |  | 146 | (0.5) |  | 419 | (1.3) |
| **Fasting glucose (mg/dL)** |  |  |  |  |  |  |  |  |  |  |  |  |  |  |  |  |  |  |  |  |  |  |  |  |  |  |  |
| <126 | 412,545 | (100.0) |  | 326513 | (79.1) |  | 57621 | (14.0) |  | 23517 | (5.7) |  | 34104 | (8.3) |  |  | 12033 | (2.9) |  | 16378 | (4.0) |  | 3043 | (0.7) |  | 13335 | (3.2) |
| ≧126 | 17,725 | (100.0) |  | 12,754 | (72.0) |  | 2,688 | (15.2) |  | 990 | (5.6) |  | 1698 | (9.6) |  |  | 1,113 | (6.3) |  | 1,170 | (6.6) |  | 264 | (1.5) |  | 906 | (5.1) |
| **Systolic blood pressure** |  |  |  |  |  |  |  |  |  |  |  |  |  |  |  |  |  |  |  |  |  |  |  |  |  |  |  |
| <140 | 371,132 | (100.0) |  | 295215 | (79.5) |  | 51478 | (13.9) |  | 20716 | (5.6) |  | 30762 | (8.3) |  |  | 10527 | (2.8) |  | 13912 | (3.7) |  | 2479 | (0.7) |  | 11433 | (3.1) |
| ≧140 | 59,138 | (100.0) |  | 44,052 | (74.5) |  | 8,831 | (14.9) |  | 3,791 | (6.4) |  | 5040 | (8.5) |  |  | 2,619 | (4.4) |  | 3,636 | (6.1) |  | 828 | (1.4) |  | 2808 | (4.7) |
| **Total cholesterol (mg/dL)** |  |  |  |  |  |  |  |  |  |  |  |  |  |  |  |  |  |  |  |  |  |  |  |  |  |  |  |
| <150 | 42,137 | (100.0) |  | 34,807 | (82.6) |  | 4,684 | (11.1) |  | 1,733 | (4.1) |  | 2951 | (7.0) |  |  | 1,353 | (3.2) |  | 1,293 | (3.1) |  | 195 | (0.5) |  | 1098 | (2.6) |
| >150 | 387,871 | (100.0) |  | 304,265 | (78.4) |  | 55,586 | (14.3) |  | 22,756 | (5.9) |  | 32830 | (8.5) |  |  | 11,779 | (3.0) |  | 16,241 | (4.2) |  | 3111 | (0.8) |  | 13130 | (3.4) |
| **High-density lipoprotein** |  |  |  |  |  |  |  |  |  |  |  |  |  |  |  |  |  |  |  |  |  |  |  |  |  |  |  |
| <35 | 358,907 | (100.0) |  | 285,983 | (79.7) |  | 48,592 | (13.5) |  | 20,879 | (5.8) |  | 27713 | (7.7) |  |  | 10,186 | (2.8) |  | 14,146 | (3.9) |  | 2,829 | (0.8) |  | 11,317 | (3.2) |
| ≧35 | 41,631 | (100.0) |  | 28,629 | (68.8) |  | 8,529 | (20.5) |  | 2,552 | (6.1) |  | 5977 | (14.4) |  |  | 2,285 | (5.5) |  | 2,188 | (5.3) |  | 293 | (0.7) |  | 1,895 | (4.6) |
| **Low-density lipoprotein** |  |  |  |  |  |  |  |  |  |  |  |  |  |  |  |  |  |  |  |  |  |  |  |  |  |  |  |
| <160 | 353,737 | (100.0) |  | 279473 | (79.0) |  | 49359 | (14.0) |  | 20216 | (5.7) |  | 29143 | (8.2) |  |  | 10663 | (3.0) |  | 14242 | (4.0) |  | 2636 | (0.7) |  | 11606 | (3.3) |
| ≧160 | 44,267 | (100.0) |  | 33,463 | (75.6) |  | 7,312 | (16.5) |  | 3,061 | (6.9) |  | 4251 | (9.6) |  |  | 1,721 | (3.9) |  | 1,771 | (4.0) |  | 451 | (1.0) |  | 1320 | (3.0) |
| **Triglycerides** |  |  |  |  |  |  |  |  |  |  |  |  |  |  |  |  |  |  |  |  |  |  |  |  |  |  |  |
| <200 | 384,047 | (100.0) |  | 308846 | (80.4) |  | 51407 | (13.4) |  | 21963 | (5.7) |  | 29444 | (7.7) |  |  | 11039 | (2.9) |  | 12755 | (3.3) |  | 2613 | (0.7) |  | 10142 | (2.6) |
| ≧200 | 45,974 | (100.0) |  | 30,230 | (65.8) |  | 8,867 | (19.3) |  | 2,531 | (5.5) |  | 6336 | (13.8) |  |  | 2,094 | (4.6) |  | 4,783 | (10.4) |  | 694 | (1.5) |  | 4089 | (8.9) |
| **Proteinuria** |  |  |  |  |  |  |  |  |  |  |  |  |  |  |  |  |  |  |  |  |  |  |  |  |  |  |  |
| Normal | 381,677 | (100.0) |  | 300447 | (78.7) |  | 54610 | (14.3) |  | 22299 | (5.8) |  | 32311 | (8.5) |  |  | 11403 | (3.0) |  | 15217 | (4.0) |  | 2900 | (0.8) |  | 12317 | (3.2) |
| Minimal proteinuria | 26,924 | (100.0) |  | 19,494 | (72.4) |  | 4,250 | (15.8) |  | 1,360 | (5.1) |  | 2890 | (10.7) |  |  | 1,213 | (4.5) |  | 1,967 | (7.3) |  | 302 | (1.1) |  | 1665 | (6.2) |
| Overt proteinuria | 2,402 | (100.0) |  | 1,747 | (72.7) |  | 299 | (12.4) |  | 103 | (4.3) |  | 196 | (8.2) |  |  | 223 | (9.3) |  | 133 | (5.5) |  | 32 | (1.3) |  | 101 | (4.2) |
| **Uric acid level** |  |  |  |  |  |  |  |  |  |  |  |  |  |  |  |  |  |  |  |  |  |  |  |  |  |  |  |
| < 7 | 318,780 | (100.0) |  | 263077 | (82.5) |  | 37974 | (11.9) |  | 16737 | (5.3) |  | 21237 | (6.7) |  |  | 8386 | (2.6) |  | 9343 | (2.9) |  | 1807 | (0.6) |  | 7536 | (2.4) |
| ≧7 | 111,490 | (100.0) |  | 76,190 | (68.3) |  | 22,335 | (20.0) |  | 7,770 | (7.0) |  | 14565 | (13.1) |  |  | 4,760 | (4.3) |  | 8,205 | (7.4) |  | 1,500 | (1.3) |  | 6,705 | (6.0) |
| **C-reactive protein (mg/L)** |  |  |  |  |  |  |  |  |  |  |  |  |  |  |  |  |  |  |  |  |  |  |  |  |  |  |  |
| <1 | 294,988 | (100.0) |  | 234540 | (79.5) |  | 41314 | (14.0) |  | 17235 | (5.8) |  | 24079 | (8.2) |  |  | 8027 | (2.7) |  | 11107 | (3.8) |  | 2150 | (0.7) |  | 8957 | (3.0) |
| 1-2.9 | 77,559 | (100.0) |  | 59,799 | (77.1) |  | 11,182 | (14.4) |  | 4,303 | (5.5) |  | 6879 | (8.9) |  |  | 2,797 | (3.6) |  | 3,781 | (4.9) |  | 692 | (0.9) |  | 3089 | (4.0) |
| 3-9.9 | 34,323 | (100.0) |  | 26,111 | (76.1) |  | 4,931 | (14.4) |  | 1,800 | (5.2) |  | 3131 | (9.1) |  |  | 1,491 | (4.3) |  | 1,790 | (5.2) |  | 314 | (0.9) |  | 1476 | (4.3) |
| ≧10 | 11,711 | (100.0) |  | 8,628 | (73.7) |  | 1,777 | (15.2) |  | 680 | (5.8) |  | 1097 | (9.4) |  |  | 653 | (5.6) |  | 653 | (5.6) |  | 101 | (0.9) |  | 552 | (4.7) |

**Table S2. Mortality risks by alcohol drinking status, compared to non-drinker**

|  |  |  | **Non-Drinker** | |  | **Modest Drinker** | | | |  | **Ex Drinker** | | | |  | **Regular Drinker** | | | |
| --- | --- | --- | --- | --- | --- | --- | --- | --- | --- | --- | --- | --- | --- | --- | --- | --- | --- | --- | --- |
|  |  |  | (n=339,267) | |  | (n=64,129) | | | |  | (n=13,146) | | | |  | (n=17,548) | | | |
|  | **ICD 9** | **Total Deaths** | **deaths** | **HRs** |  | **deaths** | **HRs** | **95%CI** | |  | **deaths** | **HRs** | **95%CI** | |  | **deaths** | **HRs** | **95%CI** | |
| **All Cause** | 001-998 | 11031 | 7156 | 1.00 |  | 1947 | 0.92 | (0.86 | , 0.97) |  | 1035 | 1.43 | (1.33 | , 1.54) |  | 893 | 1.44 | (1.33 | , 1.56) |
| Male |  | 6931 | 3453 | 1.00 |  | 1689 | 0.92 | (0.86 | , 0.98) |  | 940 | 1.46 | (1.35 | , 1.58) |  | 849 | 1.44 | (1.33 | , 1.57) |
| Female |  | 4100 | 3703 | 1.00 |  | 258 | 0.93 | (0.80 | , 1.07) |  | 95 | 1.32 | (1.05 | , 1.65) |  | 44 | 1.58 | (1.14 | , 2.19) |
| **All Cancer** | 140-208 | 4472 | 2876 | 1.00 |  | 831 | 0.97 | (0.89 | , 1.06) |  | 377 | 1.45 | (1.29 | , 1.63) |  | 388 | 1.60 | (1.42 | , 1.81) |
| Male |  | 2772 | 1333 | 1.00 |  | 723 | 0.99 | (0.90 | , 1.10) |  | 345 | 1.49 | (1.31 | , 1.69) |  | 371 | 1.63 | (1.44 | , 1.85) |
| Female |  | 1700 | 1543 | 1.00 |  | 108 | 0.88 | (0.70 | , 1.10) |  | 32 | 1.21 | (0.82 | , 1.78) |  | 17 | 1.42 | (0.83 | , 2.43) |
| Oral cancer | 140-149 | 119 | 32 | 1.00 |  | 35 | 2.37 | (1.39 | , 4.05) |  | 16 | 3.88 | (2.00 | , 7.53) |  | 36 | 5.21 | (2.96 | , 9.17) |
| Oesophagus | 150 | 98 | 15 | 1.00 |  | 26 | 4.09 | (2.02 | , 8.30) |  | 9 | 4.71 | (1.90 | 11.71 |  | 48 | 20.09 | (10.03 | 40.24) |
| Stomach | 151 | 285 | 196 | 1.00 |  | 50 | 0.90 | (0.63 | , 1.27) |  | 24 | 1.24 | (0.77 | , 2.00) |  | 15 | 1.15 | (0.66 | , 2.01) |
| Colon Rectum | 153-154 | 458 | 318 | 1.00 |  | 85 | 0.98 | (0.74 | , 1.30) |  | 35 | 1.31 | (0.89 | , 1.92) |  | 20 | 0.99 | (0.61 | , 1.60) |
| Liver | 155 | 961 | 545 | 1.00 |  | 192 | 1.07 | (0.89 | , 1.29) |  | 126 | 2.14 | (1.72 | , 2.66) |  | 98 | 1.85 | (1.45 | , 2.35) |
| Pancreas | 157 | 184 | 123 | 1.00 |  | 36 | 1.25 | (0.81 | , 1.92) |  | 8 | 1.03 | (0.49 | , 2.18) |  | 17 | 2.15 | (1.16 | , 3.96) |
| Lung | 162 | 936 | 587 | 1.00 |  | 181 | 0.85 | (0.70 | , 1.03) |  | 86 | 1.38 | (1.09 | , 1.77) |  | 82 | 1.33 | (1.02 | , 1.72) |
| Breast | 174 | 179 | 166 | 1.00 |  | 9 | 0.67 | (0.31 | , 1.44) |  | 3 | 1.70 | (0.53 | , 5.43) |  | 1 | 0.81 | (0.11 | , 6.01) |
| Prostate | 185 | 104 | 51 | 1.00 |  | 31 | 1.21 | (0.75 | , 1.94) |  | 18 | 2.16 | (1.24 | , 3.77) |  | 4 | 0.37 | (0.09 | , 1.52) |
| Bladder | 188 | 63 | 38 | 1.00 |  | 17 | 1.13 | (0.58 | , 2.20) |  | 4 | 0.79 | (0.27 | , 2.30) |  | 4 | 0.82 | (0.24 | , 2.81) |
| **Diabetes mellitus** | 250 | 672 | 498 | 1.00 |  | 85 | 0.66 | (0.50 | , 0.86) |  | 57 | 0.85 | (0.62 | , 1.18) |  | 32 | 0.75 | (0.50 | , 1.13) |
| **Cardiovascular disease** | 390-459 | 2115 | 1376 | 1.00 |  | 370 | 0.92 | (0.81 | , 1.05) |  | 235 | 1.58 | (1.35 | , 1.84) |  | 134 | 1.14 | (0.93 | , 1.39) |
| Ischemic heart disease | 410-414 | 594 | 374 | 1.00 |  | 110 | 0.94 | (0.73 | , 1.19) |  | 69 | 1.46 | (1.09 | , 1.95) |  | 41 | 1.23 | (0.86 | , 1.76) |
| Stroke | 430-438 | 874 | 571 | 1.00 |  | 148 | 0.91 | (0.74 | , 1.12) |  | 101 | 1.68 | (1.32 | , 2.13) |  | 54 | 1.06 | (0.77 | , 1.46) |
| **Expanded CVD** |  | 3064 | 2073 | 1.00 |  | 494 | 0.86 | (0.76 | , 0.96) |  | 323 | 1.35 | (1.18 | , 1.55) |  | 174 | 0.99 | (0.83 | , 1.18) |
| **Respiratory system** | 460-519 | 645 | 420 | 1.00 |  | 106 | 0.74 | (0.58 | , 0.94) |  | 77 | 1.23 | (0.93 | , 1.62) |  | 42 | 1.26 | (0.89 | , 1.78) |
| COPD | 491-496 | 256 | 160 | 1.00 |  | 43 | 0.68 | (0.46 | , 0.99) |  | 40 | 1.36 | (0.91 | , 2.03) |  | 13 | 0.79 | (0.42 | , 1.50) |
| **Digestive system** | 520-579 | 731 | 440 | 1.00 |  | 111 | 1.00 | (0.79 | , 1.27) |  | 80 | 2.03 | (1.54 | , 2.67) |  | 100 | 2.91 | (2.23 | , 3.80) |
| cirrhosis | 571 | 395 | 227 | 1.00 |  | 59 | 1.01 | (0.72 | , 1.40) |  | 44 | 2.34 | (1.62 | , 3.38) |  | 65 | 3.57 | (2.53 | , 5.03) |
| **Kidney disease** | 580-589 | 277 | 199 | 1.00 |  | 39 | 0.77 | (0.51 | , 1.15) |  | 31 | 1.06 | (0.67 | , 1.68) |  | 8 | 0.47 | (0.20 | , 1.08) |
| **Accidents** | 800-949 | 681 | 396 | 1.00 |  | 151 | 1.16 | (0.94 | , 1.44) |  | 48 | 1.51 | (1.10 | , 2.09) |  | 86 | 2.04 | (1.56 | , 2.66) |
| **Suicide** | 950-959 | 299 | 202 | 1.00 |  | 44 | 0.64 | (0.44 | , 0.94) |  | 26 | 1.97 | (1.24 | , 3.12) |  | 27 | 1.22 | (0.77 | , 1.93) |

Hazard ratios are adjusted for age, gender, education, body mass index, smoking, physical activity, systolic blood pressure, fasting blood glucose, anemia, total cholesterol, high-density lipoprotein cholesterol, low-density lipoprotein cholesterol, triglycerides, proteinuria, uric acid level and C-reactive protein in a multivariate Cox model when appropriate. Expanded CVD: CVD plus diabetes plus kidney disease.

**S3 Table .** Male demographics and clinical characteristics by drinking status

|  | **Total** | |  | **Never Drinker** | |  | **Modest Drinker** | | | | | | | |  | **Regular Drinker** | | | | | | | |
| --- | --- | --- | --- | --- | --- | --- | --- | --- | --- | --- | --- | --- | --- | --- | --- | --- | --- | --- | --- | --- | --- | --- | --- |
|  |  |  |  |  |  |  |  | |  | Never smoker | |  | Smoker | |  |  | |  | Never smoker | |  | Smoker | |
|  | n | (%) |  | n | (%) |  | n | (%) |  | n | (%) |  | n | (%) |  | n | (%) |  | n | (%) |  | n | (%) |
| **Total** | 210,594 | (100.0) |  | 137,664 | (65.4) |  | 47,540 | (22.6) |  | 14,999 | (7.1) |  | 32,541 | (15.5) |  | 25,390 | (12.1) |  | 5,303 | (2.5) |  | 20,087 | (9.5) |
| **Age** |  |  |  |  |  |  |  |  |  |  |  |  |  |  |  |  |  |  |  |  |  |  |  |
| 20-39 | 119,861 | (100.0) |  | 85,286 | (71.2) |  | 23,847 | (19.9) |  | 7,077 | (5.9) |  | 16770 | (14.0) |  | 10,728 | (9.0) |  | 1,800 | (1.5) |  | 8,928 | (7.4) |
| 40-64 | 75,502 | (100.0) |  | 43,360 | (57.4) |  | 20,052 | (26.6) |  | 6,818 | (9.0) |  | 13234 | (17.5) |  | 12,090 | (16.0) |  | 2,872 | (3.8) |  | 9,218 | (12.2) |
| ≧65 | 15,231 | (100.0) |  | 9,018 | (59.2) |  | 3,641 | (23.9) |  | 1,104 | (7.2) |  | 2537 | (16.7) |  | 2572 | (16.9) |  | 631 | (4.1) |  | 1941 | (12.7) |
| **Education** |  |  |  |  |  |  |  |  |  |  |  |  |  |  |  |  |  |  |  |  |  |  |  |
| ≦Middle school | 39,337 | (100.0) |  | 19,984 | (50.8) |  | 10,864 | (27.6) |  | 3,020 | (7.7) |  | 7844 | (19.9) |  | 8,489 | (21.6) |  | 1,539 | (3.9) |  | 6,950 | (17.7) |
| ≧High school | 169,103 | (100.0) |  | 116,415 | (68.8) |  | 36,149 | (21.4) |  | 11,807 | (7.0) |  | 24342 | (14.4) |  | 16,539 | (9.8) |  | 3,685 | (2.2) |  | 12,854 | (7.6) |
| **BMI** |  |  |  |  |  |  |  |  |  |  |  |  |  |  |  |  |  |  |  |  |  |  |  |
| <18.5 | 8,492 | (100.0) |  | 6,052 | (71.3) |  | 1,511 | (17.8) |  | 333 | (3.9) |  | 1178 | (13.9) |  | 929 | (10.9) |  | 132 | (1.6) |  | 797 | (9.4) |
| 23-30 | 192,929 | (100.0) |  | 125,684 | (65.1) |  | 44,075 | (22.8) |  | 14,063 | (7.3) |  | 30012 | (15.6) |  | 23,170 | (12.0) |  | 4,877 | (2.5) |  | 18,293 | (9.5) |
| ≧30 | 9,173 | (100.0) |  | 5,928 | (64.6) |  | 1,954 | (21.3) |  | 603 | (6.6) |  | 1351 | (14.7) |  | 1291 | (14.1) |  | 294 | (3.2) |  | 997 | (10.9) |
| **Smoking status** |  |  |  |  |  |  |  |  |  |  |  |  |  |  |  |  |  |  |  |  |  |  |  |
| Never smoker | 101,910 | (100.0) |  | 81608 | (80.1) |  | 14999 | (14.7) |  |  |  |  |  |  |  | 5303 | (5.2) |  |  |  |  |  |  |
| Smoker | 107,445 | (100.0) |  | 54,817 | (51.0) |  | 32,541 | (30.3) |  |  |  |  |  |  |  | 20,087 | (18.7) |  |  |  |  |  |  |
| **Physical activity** |  |  |  |  |  |  |  |  |  |  |  |  |  |  |  |  |  |  |  |  |  |  |  |
| Inactive | 96,426 | (100.0) |  | 63,448 | (65.8) |  | 19,931 | (20.7) |  | 5,239 | (5.4) |  | 14692 | (15.2) |  | 13,047 | (13.5) |  | 2,223 | (2.3) |  | 10,824 | (11.2) |
| Low active | 49,049 | (100.0) |  | 32,953 | (67.2) |  | 11,396 | (23.2) |  | 3,788 | (7.7) |  | 7608 | (15.5) |  | 4,700 | (9.6) |  | 982 | (2.0) |  | 3,718 | (7.6) |
| Fully active | 63,376 | (100.0) |  | 40,242 | (63.5) |  | 15,753 | (24.9) |  | 5,840 | (9.2) |  | 9913 | (15.6) |  | 7,381 | (11.6) |  | 2,037 | (3.2) |  | 5,344 | (8.4) |
| **Anemia** |  |  |  |  |  |  |  |  |  |  |  |  |  |  |  |  |  |  |  |  |  |  |  |
| No | 204,861 | (100.0) |  | 134018 | (65.4) |  | 46471 | (22.7) |  | 14681 | (7.2) |  | 31790 | (15.5) |  | 24372 | (11.9) |  | 5071 | (2.5) |  | 19301 | (9.4) |
| Yes | 5,733 | (100.0) |  | 3,646 | (63.6) |  | 1,069 | (18.6) |  | 318 | (5.5) |  | 751 | (13.1) |  | 1018 | (17.8) |  | 232 | (4.0) |  | 786 | (13.7) |
| **Systolic blood pressure** |  |  |  |  |  |  |  |  |  |  |  |  |  |  |  |  |  |  |  |  |  |  |  |
| <140 | 178,984 | (100.0) |  | 119180 | (66.6) |  | 40042 | (22.4) |  | 12372 | (6.9) |  | 27670 | (15.5) |  | 19762 | (11.0) |  | 3900 | (2.2) |  | 15862 | (8.9) |
| ≧140 | 31,610 | (100.0) |  | 18,484 | (58.5) |  | 7,498 | (23.7) |  | 2,627 | (8.3) |  | 4871 | (15.4) |  | 5,628 | (17.8) |  | 1403 | (4.4) |  | 4225 | (13.4) |
| **Fasting glucose (mg/dL)** |  |  |  |  |  |  |  |  |  |  |  |  |  |  |  |  |  |  |  |  |  |  |  |
| <126 | 200,743 | (100.0) |  | 132204 | (65.9) |  | 45201 | (22.5) |  | 14299 | (7.1) |  | 30902 | (15.4) |  | 23338 | (11.6) |  | 4811 | (2.4) |  | 18527 | (9.2) |
| ≧126 | 9,851 | (100.0) |  | 5,460 | (55.4) |  | 2,339 | (23.7) |  | 700 | (7.1) |  | 1639 | (16.6) |  | 2,052 | (20.8) |  | 492 | (5.0) |  | 1560 | (15.8) |
| **Total cholesterol (mg/dL)** | |  |  |  |  |  |  |  |  |  |  |  |  |  |  |  |  |  |  |  |  |  |  |
| <150 | 18,582 | (100.0) |  | 12,997 | (69.9) |  | 3,498 | (18.8) |  | 957 | (5.2) |  | 2541 | (13.7) |  | 2,087 | (11.2) |  | 420 | (2.3) |  | 1667 | (9.0) |
| >150 | 191,851 | (100.0) |  | 124,557 | (64.9) |  | 44,012 | (22.9) |  | 14,031 | (7.3) |  | 29981 | (15.6) |  | 23,282 | (12.1) |  | 4879 | (2.5) |  | 18403 | (9.6) |
| **High-density lipoprotein** | |  |  |  |  |  |  |  |  |  |  |  |  |  |  |  |  |  |  |  |  |  |  |
| <35 | 162,772 | (100.0) |  | 106,369 | (65.3) |  | 36,909 | (22.7) |  | 12,130 | (7.5) |  | 24779 | (15.2) |  | 19,494 | (12.0) |  | 4,238 | (2.6) |  | 15,256 | (9.4) |
| ≧35 | 31,272 | (100.0) |  | 19,159 | (61.3) |  | 7,919 | (25.3) |  | 2,123 | (6.8) |  | 5796 | (18.5) |  | 4,194 | (13.4) |  | 750 | (2.4) |  | 3,444 | (11.0) |
| **Low-density lipoprotein** |  |  |  |  |  |  |  |  |  |  |  |  |  |  |  |  |  |  |  |  |  |  |  |
| <160 | 168,004 | (100.0) |  | 109364 | (65.1) |  | 38347 | (22.8) |  | 12060 | (7.2) |  | 26287 | (15.6) |  | 20293 | (12.1) |  | 4223 | (2.5) |  | 16070 | (9.6) |
| ≧160 | 24,532 | (100.0) |  | 15,432 | (62.9) |  | 6,086 | (24.8) |  | 2,083 | (8.5) |  | 4003 | (16.3) |  | 3,014 | (12.3) |  | 723 | (2.9) |  | 2291 | (9.3) |
| **Triglycerides** |  |  |  |  |  |  |  |  |  |  |  |  |  |  |  |  |  |  |  |  |  |  |  |
| <200 | 177,669 | (100.0) |  | 119364 | (67.2) |  | 39306 | (22.1) |  | 12953 | (7.3) |  | 26353 | (14.8) |  | 18999 | (10.7) |  | 4265 | (2.4) |  | 14734 | (8.3) |
| ≧200 | 32,808 | (100.0) |  | 18,226 | (55.6) |  | 8,206 | (25.0) |  | 2,038 | (6.2) |  | 6168 | (18.8) |  | 6,376 | (19.4) |  | 1034 | (3.2) |  | 5342 | (16.3) |
| **Proteinuria** |  |  |  |  |  |  |  |  |  |  |  |  |  |  |  |  |  |  |  |  |  |  |  |
| Normal | 194,692 | (100.0) |  | 128670 | (66.1) |  | 43742 | (22.5) |  | 14071 | (7.2) |  | 29671 | (15.2) |  | 22280 | (11.4) |  | 4756 | (2.4) |  | 17524 | (9.0) |
| Minimal proteinuria | 14,404 | (100.0) |  | 8,141 | (56.5) |  | 3,511 | (24.4) |  | 855 | (5.9) |  | 2656 | (18.4) |  | 2,752 | (19.1) |  | 468 | (3.2) |  | 2284 | (15.9) |
| Overt proteinuria | 1,298 | (100.0) |  | 728 | (56.1) |  | 252 | (19.4) |  | 67 | (5.2) |  | 185 | (14.3) |  | 318 | (24.5) |  | 68 | (5.2) |  | 250 | (19.3) |
| **Uric acid level** |  |  |  |  |  |  |  |  |  |  |  |  |  |  |  |  |  |  |  |  |  |  |  |
| < 7 | 116,780 | (100.0) |  | 77572 | (66.4) |  | 26188 | (22.4) |  | 7988 | (6.8) |  | 18200 | (15.6) |  | 13020 | (11.1) |  | 2609 | (2.2) |  | 10411 | (8.9) |
| ≧7 | 93,814 | (100.0) |  | 60,092 | (64.1) |  | 21,352 | (22.8) |  | 7,011 | (7.5) |  | 14341 | (15.3) |  | 12,370 | (13.2) |  | 2,694 | (2.9) |  | 9,676 | (10.3) |
| **C-reactive protein (mg/L)** | |  |  |  |  |  |  |  |  |  |  |  |  |  |  |  |  |  |  |  |  |  |  |
| <1 | 142,118 | (100.0) |  | 94330 | (66.4) |  | 32180 | (22.6) |  | 10442 | (7.3) |  | 21738 | (15.3) |  | 15608 | (11.0) |  | 3376 | (2.4) |  | 12232 | (8.6) |
| 1-2.9 | 39,436 | (100.0) |  | 24,724 | (62.7) |  | 9,105 | (23.1) |  | 2,739 | (6.9) |  | 6366 | (16.1) |  | 5,607 | (14.2) |  | 1132 | (2.9) |  | 4475 | (11.3) |
| 3-9.9 | 17,009 | (100.0) |  | 10,266 | (60.4) |  | 3,994 | (23.5) |  | 1,103 | (6.5) |  | 2891 | (17.0) |  | 2,749 | (16.2) |  | 527 | (3.1) |  | 2222 | (13.1) |
| ≧10 | 6,193 | (100.0) |  | 3,626 | (58.5) |  | 1,440 | (23.3) |  | 416 | (6.7) |  | 1024 | (16.5) |  | 1127 | (18.2) |  | 197 | (3.2) |  | 930 | (15.0) |

**S4 Table.** Female demographics and clinical characteristics by drinking status

|  | **Total** | |  | **Never Drinker** | |  | **Modest Drinker** | | | | | | | |  | **Regular Drinker** | | | | | | | |
| --- | --- | --- | --- | --- | --- | --- | --- | --- | --- | --- | --- | --- | --- | --- | --- | --- | --- | --- | --- | --- | --- | --- | --- |
|  |  |  |  |  |  |  |  | |  | Never smoker | |  | Smoker | |  |  | |  | Never smoker | |  | Smoker | |
|  | n | (%) |  | n | (%) |  | n | (%) |  | n | (%) |  | n | (%) |  | n | (%) |  | n | (%) |  | n | (%) |
| **Total** | 219,422 | (100.0) |  | 201,603 | (91.9) |  | 12,769 | (5.8) |  | 9,508 | (4.3) |  | 3,261 | (1.5) |  | 5,050 | (2.3) |  | 2,355 | (1.1) |  | 2,695 | (1.2) |
| **Age** |  |  |  |  |  |  |  |  |  |  |  |  |  |  |  |  |  |  |  |  |  |  |  |
| 20-39 | 123,862 | (100.0) |  | 114,423 | (92.4) |  | 6,631 | (5.4) |  | 4,305 | (3.5) |  | 2326 | (1.9) |  | 2,808 | (2.3) |  | 980 | (0.8) |  | 1,828 | (1.5) |
| 40-64 | 82,725 | (100.0) |  | 75,034 | (90.7) |  | 5,676 | (6.9) |  | 4,841 | (5.9) |  | 835 | (1.0) |  | 2,015 | (2.4) |  | 1,226 | (1.5) |  | 789 | (1.0) |
| ≧65 | 12,835 | (100.0) |  | 12,146 | (94.6) |  | 462 | (3.6) |  | 362 | (2.8) |  | 100 | (0.8) |  | 227 | (1.8) |  | 149 | (1.2) |  | 78 | (0.6) |
| **Education** |  |  |  |  |  |  |  |  |  |  |  |  |  |  |  |  |  |  |  |  |  |  |  |
| ≦Middle school | 63,638 | (100.0) |  | 57,234 | (89.9) |  | 4,464 | (7.0) |  | 3,648 | (5.7) |  | 816 | (1.3) |  | 1,940 | (3.0) |  | 1,072 | (1.7) |  | 868 | (1.4) |
| ≧High school | 153,390 | (100.0) |  | 142,241 | (92.7) |  | 8,118 | (5.3) |  | 5,718 | (3.7) |  | 2400 | (1.6) |  | 3,031 | (2.0) |  | 1,239 | (0.8) |  | 1,792 | (1.2) |
| **BMI** |  |  |  |  |  |  |  |  |  |  |  |  |  |  |  |  |  |  |  |  |  |  |  |
| <18.5 | 28,586 | (100.0) |  | 26,420 | (92.4) |  | 1,417 | (5.0) |  | 813 | (2.8) |  | 604 | (2.1) |  | 749 | (2.6) |  | 221 | (0.8) |  | 528 | (1.8) |
| 23-30 | 183,582 | (100.0) |  | 168,609 | (91.8) |  | 10,937 | (6.0) |  | 8,373 | (4.6) |  | 2564 | (1.4) |  | 4,036 | (2.2) |  | 1,978 | (1.1) |  | 2,058 | (1.1) |
| ≧30 | 7,254 | (100.0) |  | 6,574 | (90.6) |  | 415 | (5.7) |  | 322 | (4.4) |  | 93 | (1.3) |  | 265 | (3.7) |  | 156 | (2.2) |  | 109 | (1.5) |
| **Smoking status** |  |  |  |  |  |  |  |  |  |  |  |  |  |  |  |  |  |  |  |  |  |  |  |
| Never smoker | 197,634 | (100.0) |  | 185771 | (94.0) |  | 9508 | (4.8) |  |  |  |  |  |  |  | 2355 | (1.2) |  |  |  |  |  |  |
| Smoker | 17,984 | (100.0) |  | 12,028 | (66.9) |  | 3,261 | (18.1) |  |  |  |  |  |  |  | 2,695 | (15.0) |  |  |  |  |  |  |
| **Physical activity** |  |  |  |  |  |  |  |  |  |  |  |  |  |  |  |  |  |  |  |  |  |  |  |
| Inactive | 126,737 | (100.0) |  | 116,783 | (92.1) |  | 6,651 | (5.2) |  | 4,663 | (3.7) |  | 1988 | (1.6) |  | 3,303 | (2.6) |  | 1,398 | (1.1) |  | 1,905 | (1.5) |
| Low active | 48,727 | (100.0) |  | 44,666 | (91.7) |  | 3,214 | (6.6) |  | 2,439 | (5.0) |  | 775 | (1.6) |  | 847 | (1.7) |  | 412 | (0.8) |  | 435 | (0.9) |
| Fully active | 41,893 | (100.0) |  | 38,326 | (91.5) |  | 2,739 | (6.5) |  | 2,270 | (5.4) |  | 469 | (1.1) |  | 828 | (2.0) |  | 502 | (1.2) |  | 326 | (0.8) |
| **Anemia** |  |  |  |  |  |  |  |  |  |  |  |  |  |  |  |  |  |  |  |  |  |  |  |
| No | 193,966 | (100.0) |  | 177956 | (91.7) |  | 11448 | (5.9) |  | 8450 | (4.4) |  | 2998 | (1.5) |  | 4562 | (2.4) |  | 2078 | (1.1) |  | 2484 | (1.3) |
| Yes | 25,456 | (100.0) |  | 23,647 | (92.9) |  | 1,321 | (5.2) |  | 1,058 | (4.2) |  | 263 | (1.0) |  | 488 | (1.9) |  | 277 | (1.1) |  | 211 | (0.8) |
| **Systolic blood pressure** |  |  |  |  |  |  |  |  |  |  |  |  |  |  |  |  |  |  |  |  |  |  |  |
| <140 | 191,953 | (100.0) |  | 176035 | (91.7) |  | 11436 | (6.0) |  | 8344 | (4.3) |  | 3092 | (1.6) |  | 4482 | (2.3) |  | 1972 | (1.0) |  | 2510 | (1.3) |
| ≧140 | 27,469 | (100.0) |  | 25,568 | (93.1) |  | 1,333 | (4.9) |  | 1,164 | (4.2) |  | 169 | (0.6) |  | 568 | (2.1) |  | 383 | (1.4) |  | 185 | (0.7) |
| **Fasting glucose (mg/dL)** |  |  |  |  |  |  |  |  |  |  |  |  |  |  |  |  |  |  |  |  |  |  |  |
| <126 | 211,575 | (100.0) |  | 194309 | (91.8) |  | 12420 | (5.9) |  | 9218 | (4.4) |  | 3202 | (1.5) |  | 4846 | (2.3) |  | 2231 | (1.1) |  | 2615 | (1.2) |
| ≧126 | 7,847 | (100.0) |  | 7,294 | (93.0) |  | 349 | (4.4) |  | 290 | (3.7) |  | 59 | (0.8) |  | 204 | (2.6) |  | 124 | (1.6) |  | 80 | (1.0) |
| **Total cholesterol (mg/dL)** | |  |  |  |  |  |  |  |  |  |  |  |  |  |  |  |  |  |  |  |  |  |  |
| <150 | 23,533 | (100.0) |  | 21,810 | (92.7) |  | 1,186 | (5.0) |  | 776 | (3.3) |  | 410 | (1.7) |  | 537 | (2.3) |  | 197 | (0.8) |  | 340 | (1.4) |
| >150 | 195,789 | (100.0) |  | 179,708 | (91.8) |  | 11,574 | (5.9) |  | 8,725 | (4.5) |  | 2849 | (1.5) |  | 4,507 | (2.3) |  | 2156 | (1.1) |  | 2351 | (1.2) |
| **High-density lipoprotein** | |  |  |  |  |  |  |  |  |  |  |  |  |  |  |  |  |  |  |  |  |  |  |
| <35 | 195,926 | (100.0) |  | 179,614 | (91.7) |  | 11,683 | (6.0) |  | 8,749 | (4.5) |  | 2934 | (1.5) |  | 4,629 | (2.4) |  | 2,178 | (1.1) |  | 2,451 | (1.3) |
| ≧35 | 10,322 | (100.0) |  | 9,470 | (91.7) |  | 610 | (5.9) |  | 429 | (4.2) |  | 181 | (1.8) |  | 242 | (2.3) |  | 94 | (0.9) |  | 148 | (1.4) |
| **Low-density lipoprotein** |  |  |  |  |  |  |  |  |  |  |  |  |  |  |  |  |  |  |  |  |  |  |  |
| <160 | 185,519 | (100.0) |  | 170109 | (91.7) |  | 11012 | (5.9) |  | 8156 | (4.4) |  | 2856 | (1.5) |  | 4398 | (2.4) |  | 2023 | (1.1) |  | 2375 | (1.3) |
| ≧160 | 19,705 | (100.0) |  | 18,031 | (91.5) |  | 1,226 | (6.2) |  | 978 | (5.0) |  | 248 | (1.3) |  | 448 | (2.3) |  | 239 | (1.2) |  | 209 | (1.1) |
| **Triglycerides** |  |  |  |  |  |  |  |  |  |  |  |  |  |  |  |  |  |  |  |  |  |  |  |
| <200 | 206,165 | (100.0) |  | 189482 | (91.9) |  | 12101 | (5.9) |  | 9010 | (4.4) |  | 3091 | (1.5) |  | 4582 | (2.2) |  | 2126 | (1.0) |  | 2456 | (1.2) |
| ≧200 | 13,126 | (100.0) |  | 12,004 | (91.5) |  | 661 | (5.0) |  | 493 | (3.8) |  | 168 | (1.3) |  | 461 | (3.5) |  | 227 | (1.7) |  | 234 | (1.8) |
| **Proteinuria** |  |  |  |  |  |  |  |  |  |  |  |  |  |  |  |  |  |  |  |  |  |  |  |
| Normal | 186,785 | (100.0) |  | 171777 | (92.0) |  | 10868 | (5.8) |  | 8228 | (4.4) |  | 2640 | (1.4) |  | 4140 | (2.2) |  | 1976 | (1.1) |  | 2164 | (1.2) |
| Minimal proteinuria | 12,487 | (100.0) |  | 11,353 | (90.9) |  | 739 | (5.9) |  | 505 | (4.0) |  | 234 | (1.9) |  | 395 | (3.2) |  | 170 | (1.4) |  | 225 | (1.8) |
| Overt proteinuria | 1,096 | (100.0) |  | 1,019 | (93.0) |  | 47 | (4.3) |  | 36 | (3.3) |  | 11 | (1.0) |  | 30 | (2.7) |  | 16 | (1.5) |  | 14 | (1.3) |
| **Uric acid level** |  |  |  |  |  |  |  |  |  |  |  |  |  |  |  |  |  |  |  |  |  |  |  |
| < 7 | 201,809 | (100.0) |  | 185505 | (91.9) |  | 11786 | (5.8) |  | 8749 | (4.3) |  | 3037 | (1.5) |  | 4518 | (2.2) |  | 2079 | (1.0) |  | 2439 | (1.2) |
| ≧7 | 17,613 | (100.0) |  | 16,098 | (91.4) |  | 983 | (5.6) |  | 759 | (4.3) |  | 224 | (1.3) |  | 532 | (3.0) |  | 276 | (1.6) |  | 256 | (1.5) |
| **C-reactive protein (mg/L)** | |  |  |  |  |  |  |  |  |  |  |  |  |  |  |  |  |  |  |  |  |  |  |
| <1 | 152,715 | (100.0) |  | 140210 | (91.8) |  | 9134 | (6.0) |  | 6793 | (4.4) |  | 2341 | (1.5) |  | 3371 | (2.2) |  | 1523 | (1.0) |  | 1848 | (1.2) |
| 1-2.9 | 38,078 | (100.0) |  | 35,075 | (92.1) |  | 2,077 | (5.5) |  | 1,564 | (4.1) |  | 513 | (1.3) |  | 926 | (2.4) |  | 474 | (1.2) |  | 452 | (1.2) |
| 3-9.9 | 17,279 | (100.0) |  | 15,845 | (91.7) |  | 937 | (5.4) |  | 697 | (4.0) |  | 240 | (1.4) |  | 497 | (2.9) |  | 230 | (1.3) |  | 267 | (1.5) |
| ≧10 | 5,504 | (100.0) |  | 5,002 | (90.9) |  | 337 | (6.1) |  | 264 | (4.8) |  | 73 | (1.3) |  | 165 | (3.0) |  | 78 | (1.4) |  | 87 | (1.6) |
